# Proposed Unifying Classification Criteria for Spinal Nerve Root Variations

**DOI:** 10.31486/toj.21.0014

**Published:** 2021

**Authors:** Vladimir N. Nikolenko, Alexey N. Shkarubo, Ekaterina Zmeeva, Mikhail Y. Sinelnikov

**Affiliations:** ^1^ Sechenov University, Moscow, Russian Federation; ^2^ Burdenko Neurosurgical Clinic, Moscow, Russian Federation; ^3^ Research Institute of Human Morphology, Moscow, Russian Federation

## TO THE EDITOR

Existing classification systems for spinal nerve root anomalies are numerous and substantially different from one another. Spinal nerve root abnormalities are known to be congenital deformities^1^ and are perceived to be the result of incorrect migration of nerve fibers, causing structural and spatial disorganization.^2-3^ Spinal nerves are formed from the ventral and dorsal roots in the spinal canal.^4^ Advances in anatomic research have prompted updated classifications for anatomic variations, known pathologic conditions, and clinical impacts of spinal nerve roots. Stratification of knowledge of these variations and abnormalities is important to understand the pathologic changes associated with these conditions and can be achieved through formulating specific unification criteria within existing classification systems. Upon analysis of the existing, most-used classification systems, we propose unifying classification criteria.

In 1962, Cannon et al first identified the 3 most common variations of nerve root anomalies: conjoined Type I, anastomosed Type II, and transverse Type III.^1-2^ In 1982, Postacchini et al classified common spinal nerve root abnormalities into 5 different variations.^3^ In 1983, Neidre and MacNab expanded Cannon's classification based on the angle and positioning of root emergence.^5^ In 1984, Kadish and Simmons introduced a classification system based on anatomic and radiologic findings.^6^ Chotigavanich and Sawangnatra provided a unified classification in 1992.^7^ The most recent (2020) classification update can be attributed to Haviarová et al.^8^

All the classifications describe similar processes (Figure). Therefore, we propose a unified classification based on type of deformity and localization (Table).

**Figure.**
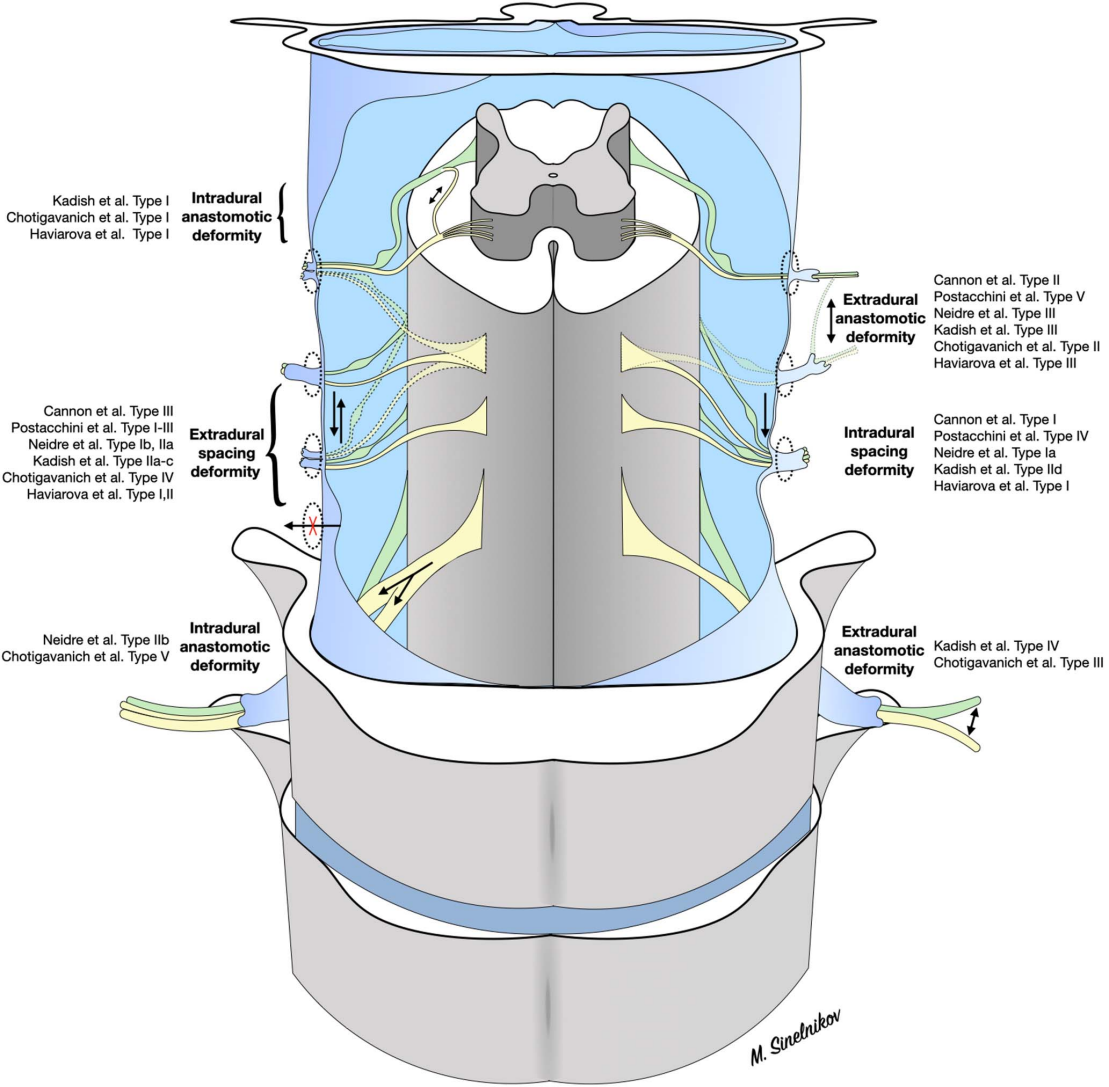
Schematic representation of unified spinal nerve root anomaly classifications.

**Table.** Existing Classifications of Spinal Nerve Root Anomalies Arranged by Unifying Criteria

	Unifying Criteria			
Existing Classification Reference	Intradural Anastomotic Deformity	Intradural Spacing Deformity	Extradural Anastomotic Deformity	Extradural Spacing Deformity
Cannon et al, 1962^2^	–	Type 1	Type II	Type III
Postacchini et al, 1982^3^	–	Type IV	Type V	Types I-III
Neidre and MacNab, 1983^5^	Type IIb	Type Ia	Type III	Types Ib, IIa
Kadish and Simmons, 1984^6^	Type I	Type IId	Types III, IV	Types IIa-c
Chotigavanich and Sawangnatra, 1992^7^	Type I, V	–	Type II, III	Type IV
Haviarová et al, 2020^8^	Type I	Type I	Type III	Types I, II

Based on existing classification systems for spinal nerve root anomalies, certain unifying criteria can be extracted. Primarily, all classification systems focus on intradural and extradural anastomoses. Further, all classification systems include descriptions of different anastomotic pathologies (intradural anastomoses, extradural anastomoses) and spacing deformities (aberrant root, transverse root, conjoined roots, caudal root). As such, the unifying criteria for spinal nerve root variations can be separated into the 2 main groups of intradural and extradural, each of which has 2 types of pathologies: anastomotic deformities and spacing deformities (Table). We therefore propose 4 variation types: intradural anastomotic deformities, intradural spacing deformities, extradural anastomotic deformities, and extradural spacing deformities. These 4 variations include all previously classified pathology types and can serve as a unifying classification system. Such a unification is warranted by the significantly different existing classification systems that complicate data stratification, focused research, and reporting. The abundance of different diagnostic criteria is associated with certain diagnostic and clinical struggles. Medical science always strives toward unification, as it provides a substantial basis for further research.
